# How to Alleviate Feelings of Crowding in a Working from Home Environment: Lessons Learned from the COVID-19 Pandemic

**DOI:** 10.3390/ijerph20021025

**Published:** 2023-01-06

**Authors:** So Yeon Park, Caroline Newton, Rachel Lee

**Affiliations:** 1 Department of Urbanism, Faculty of Architecture and the Built Environment, Delft University of Technology (TU Delft), Julianalaan 134, 2628BL Delft, The Netherlands; 2 Department of Architecture, Faculty of Architecture and the Built Environment, Delft University of Technology (TU Delft), Julianalaan 134, 2628BL Delft, The Netherlands

**Keywords:** feelings of crowding, working from home, work environment, COVID-19, health, productivity

## Abstract

The sudden adoption of working from home (WFH) during the COVID-19 pandemic has required the reconfiguration of home spaces to fit space for remote work into existing spaces already filled with other domestic functions. This resulted in blurring of home and work boundaries, the potential lack of space for telecommuting from home, and telecommuters’ feelings of crowding. Numerous studies have shown the negative effects of crowding feelings on workers’ responses. This study focused on the issue of crowding in the residential workspace. An online survey was conducted to investigate how features of the home workspace correlate with telecommuters’ feelings of crowding and how these feelings affect satisfaction, health, and productivity. As a result, we found that various environmental features of home workspaces (e.g., house size, purpose of workspace, accessible balcony, lighting, noise, etc.), as well as psychological aspects (e.g., individual control over space use), had significant effects on telecommuters’ feelings of crowdedness. It was also found that feelings of crowding in the WFH environment can directly and indirectly affect teleworkers’ satisfaction with work environments, well-being, and work performance. Based on the results, we offered various potential ways to alleviate overcrowding issues in the WFH context.

## 1. Introduction

Since the outbreak of coronavirus disease in 2019 (COVID-19), everyone’s daily lives have changed dramatically. Many daily activities conducted in public spaces, such as working, meeting, learning, and socializing, have been converted to home spaces under lockdown measures worldwide. The residential environment has become increasingly important to people’s well-being, as the types of functions that a house must also accommodate have expanded rapidly.

The sudden and somewhat forced adoption of working from home (WFH) due to the COVID-19 pandemic demanded the reconfiguration of home spaces. Space for WFH had to fit into existing physical spaces, already densely filled with other domestic functions. Furthermore, many individuals had to share these spaces with family members who also worked or learned from home. In their literature review on WFH, Ammons and Markham noted that the nearly unavoidable blurring of home and work boundaries would produce a set of challenges, such as distractions and recurring conflicts between work and home duties [1]. The ambiguity of home workspace boundaries has received much attention during the COVID-19 pandemic [2,3,4]. In addition, numerous surveys and studies have cited the lack of space for WFH as a major factor negatively impacting telecommuters [5,6,7] in terms of their well-being [8,9], productivity [10,11], and satisfaction [9].

Several studies have been devoted to the effect of office space characteristics, such as noise, lighting, air quality, thermal comfort, indoor plants, and workspace layout, on the perception, behavior, and productivity of workers—e.g., [12,13,14,15,16,17]. In particular, since conventional closed workplaces started to be replaced with open-plan design in the 1970s, many researchers have focused on the issues of spatial density, interpersonal distance, and the feeling of crowding in the workplace—e.g., [18,19,20]. According to several empirical studies, high spatial density, limited privacy, and feelings of crowding negatively affect workers’ satisfaction, health, and performance [18,21,22,23,24].

Despite the abundance of evidence connecting office environments and worker experiences, it may be inappropriate to directly apply this knowledge to WFH because the WFH context has fundamentally different physical and social conditions than the office environment. In addition, the two work environments differ in how workers communicate, interact, and relax. Furthermore, the preconceived social etiquette required for each workspace is not the same. These inherent variances directly affect how dissatisfaction with workspace size, feelings of crowding, and invasion of privacy occur while working.

Interest in WFH began in the 1970s, when the cost of commuting rose sharply after the oil crisis. Since then, this interest has grown steadily and modestly until the COVID-19 pandemic. Continued advances in information and communication technologies (ICTs) have been an important driver of WFH’s expansion [25]. Accordingly, most countries with relatively high rates of WFH prior to COVID-19 ranked high on the ICT Development Index [26,27]. For example, of the 27 EU countries, the ten countries with the highest rates of WFH in 2019 (in descending order: Sweden, the Netherlands, Luxembourg, Finland, Denmark, Belgium, France, Austria, Estonia, and Ireland) were all in the top 25 in the 2017 ICT Development Index. On the other hand, interestingly, South Korea (hereafter Korea) ranked second in the 2017 ICT Development Index, but the proportion of WFH before the pandemic was exceptionally low. Kim and Ju estimated the Korean national WFH ratio in 2020 to be only 1.23%, based on WFH data from 2000 and 2010 [28]. However, unexpectedly, COVID-19 has given Korea and many other nations an “opportunity” to accept and adapt to a new work type, WFH.

Many surveys have shown that following the pandemic, most Korean workers prefer ‘hybrid work’, which allows them to work from home or the office according to their needs—e.g., [29,30]. Accordingly, several companies are designing new work arrangements that offer individual choices [31]. Considering that WFH will remain a “new normal” work type, it is crucial to evaluate how effectively the existing residential environment has accommodated WFH and to derive factors to consider when developing new housing models. The necessity for such studies may be greater, especially in Korea, because Korea had limited experience with WFH before the pandemic, and the homogeneity of housing types in Korea makes it difficult to flexibly accommodate residents’ diverse needs. In this regard, the present study aims to comprehend how Korean telecommuters living in Korea and the Netherlands were impacted by the features of home workspaces during COVID-19 and to recommend design suggestions to better reflect the new demands on the residential environment in the Korean context.

### 1.1. Feelings of Crowding in the Work Environment

Many studies have explored the effect of workspace size on worker productivity, satisfaction, and well-being. These studies can be classified according to whether the main research variable (indicating workspace size) is an objectively measurable spatial density (measured in square meters or meters)—e.g., [18] or a subjectively perceived density (i.e., feelings of crowding)—e.g., [32].

High spatial density in the workplace can create feelings of crowding and is significantly related to low work satisfaction, poor job performance, and high turnover intentions [18,21,22,23,24]. Other researchers have focused on interpersonal distance. Kraut et al. showed that less interpersonal distance can increase unnecessary cognitive load while working [33]. However, contradictory research suggests that the closer employees are to colleagues, the more they communicate and collaborate [33,34,35,36,37,38,39]. Other studies have examined spatial openness as another variable indicating workspace size. The number and height of partitions were found to determine how open a workspace is and to affect privacy, distraction, and satisfaction [20,24,40,41].

Crowding differs from objective numerical parameters, such as spatial density or interpersonal distance [42]. It is a subjective term that depends on an individual’s perceived degree of congestion under a given spatial density. Therefore, the feeling of crowding is affected not only by spatial density [18,43,44], but also by individuals’ characteristics, including sex [45], preference for interpersonal distance [46], and cultural background [47,48].

The physical and social features of an environment also influence feelings of crowding. For example, high ceilings and visual exits (e.g., windows and doors) reduce the feeling of crowding [49]. People are likely to feel overcrowded when other stressors, such as noise, are involved [50]. In addition, if the activity is carried out in the center of the room rather than in the corner, crowding is less probable [51]. When a room is bright due to its main color or appropriate lighting, crowding can be lessened [52,53,54]. In addition to brightness, people can feel less overcrowded when wall installations (e.g., paintings or advertisements) distract their attention [55,56]. Altman argued that crowding can be determined by levels of privacy. If someone’s privacy is compromised, a feeling of crowding is more likely to exist [57].

Numerous studies have demonstrated the significant impact of crowding on workers’ behavioral and psychological responses. For example, Baron indicated that crowding and a lack of privacy may promote arousal, information overload, and environmental stress, and diminish a sense of control over a space, which can influence job performance [58]. More recently, the US-based furniture company Knoll established that crowding strongly impacts employee satisfaction with the workplace [44]. Kim and de Dear also found that satisfaction with the amount of available workspace influences employees’ overall perceptions of the workspace [59]. In addition to satisfaction and productivity, crowding affects deviant workplace behaviors, such as violent and antisocial behavior [60,61]. All of these results show that how workers feel about the amount of their workspace has a profound effect on their well-being and productivity. In this sense, the present study particularly focused on the issue of crowding in the WFH context.

Several researchers have interpreted and defined crowding differently, such as social overload [62,63], behavior constraint [32,64], unwanted interaction [65,66], scarcity of resources [67], and failure to achieve the desired level of privacy [57]. The general concept of crowding in a typical office space can be associated with physical distance from co-workers, overload due to unwanted interactions, or degrees of privacy. On the other hand, crowding in a home workspace requires a different approach because the space is usually shared only with the family or is used alone and often accommodates various domestic functions. In this study, we measured the satisfaction of home workspace size as a variable representing crowding in WFH settings. This is based on the APA dictionary of psychology’s definition of crowding: “psychological tension produced… especially when individuals feel that the amount of space available to them is insufficient for their needs” [68].

### 1.2. Research Hypotheses

The feeling of crowding can be controlled not only by spatial density itself but also by lighting, noise, visual distractions, and privacy, as previously discussed [49,50,51,52,53,54,55,56,57]. This perspective allowed us to infer that various architectural modifications can be attempted when workspace size dissatisfies occupants. Our first hypothesis is related to identifying these architectural modifications.

**H1.**  *Various environmental features of the home workspace (space size, density, openness, lighting, noise, privacy, and visual distractions) are associated with telecommuters’ feelings of crowding*.

Through the results of testing the first hypothesis, we extracted various architectural design ideas for alleviating the crowding issue in the home workspace.

As previously mentioned, there is ample evidence that crowding feelings in the workplace affect workers’ perceptions and behaviors [44,58,59,60,61]. Our second and third hypotheses were determined to examine these relationships in the WFH context. The second hypothesis investigates the impact of telecommuters’ crowding feelings on their ratings for home workspace (H2), and the third hypothesis assumes the influence of crowding feelings on changes in health (H3a) and productivity (H3b) before and during COVID-19.

**H2.**  *Telecommuters’ feelings of crowding affect how they rate their home workspaces*.

**H3.**  *Telecommuters’ feelings of crowding affect changes in (a) health and (b) productivity before and during COVID-19*.

Finally, based on previous studies highlighting the importance of worker satisfaction with the work environment for workers’ well-being and performance—e.g., [69,70]—we investigated how telecommuters’ satisfaction with the home workspace affected their health (H4a) and productivity (H4b) while working from home.

**H4.**  *Telecommuters’ ratings for home workspace affect changes in (a) health and (b) productivity before and during COVID-19*.

**Figure 1:**
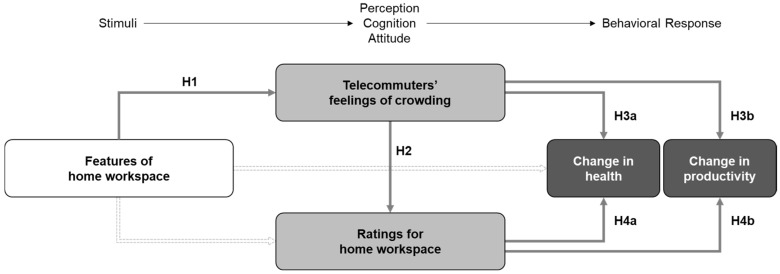
Overview of conceptual model and hypotheses.

Figure 1 shows the hypothesized relationships between the research variables substituted into the stimulus-response model. As Pang et al. found, the spatial elements of the home workspace can affect occupants’ satisfaction, well-being, and productivity [6]. However, investigating these relationships requires extensive research, and thus was excluded from the scope of this study.

## 2. Materials and Methods

### 2.1. Participants and Procedures

Apartments, indicating large-scale apartment complexes (62.3%), as well as multi-family houses (14.9%), are the most dominant housing type in Korea, accounting for 77.2% of housing units in 2019 [71]. It would be no exaggeration to say that most Koreans live in apartments (mostly developed using standardized and prototyped floor layouts). Since this study examines the occupants’ WFH experiences according to differences in living environments, it was considered that recruiting only those living in similar living environments as respondents could be problematic. To compensate for this, we recruited Koreans who had experienced WFH during COVID-19, not only in Korea but also in the Netherlands. The Netherlands, similar to Korea, has advanced ICT, an essential facility for WFH. However, the Netherlands, unlike Korea, has various types of houses, including detached, semi-detached, terraced houses, townhouses, apartments, and even houseboats. Therefore, we included two groups with the same cultural and ethnic backgrounds but different living conditions to avoid obtaining skewed responses for the independent variables.

An online survey was created using Qualtrics and approved by the Human Research Ethics Committee at TU Delft. The survey link was distributed through social networking services (e.g., Facebook, LinkedIn, and KakaoTalk) in Korea and the Netherlands from 4 November to 29 December 2021. Through the suitability check, we limited respondents to Koreans who had worked from home during the pandemic in Korea or the Netherlands and who spent a good amount of time at a desk while working. Those who solely worked from home before COVID-19 were also excluded because participants were asked to compare working in an office (or a workspace other than home) before COVID-19 with WFH during COVID-19.

Among the 253 individuals who provided informed consent and completed the survey, 190 lived in Korea, and 63 lived in the Netherlands. Of the participants, 18.6% were 21–30 years old, 65.6% were 31–40 years old, and 15.8% were 41 years or older. More women (65.2%) participated than men (34.8%), and no one responded as non-binary or third gender. Of the participants, 55.3% were married, and 2.8% were in domestic partnerships; 36.4% were single, and 2.4% were widowed or divorced. Eight participants preferred not to disclose their marital status.

### 2.2. Measures

After the suitability check, the respondents were asked to answer questions about personal information and the research variables shown in Figure 1 (i.e., features of the home workspace, feelings of crowding, ratings for the home workspace, and changes in health and productivity). The type of response was determined among multiple-choice, 5-point Likert scale, and open-ended according to the characteristics of the variables.

#### 2.2.1. Feelings of Crowding (Satisfaction with Home Workspace Size)

We considered the space occupied by telecommuters while working from home to be a separate “room” with furniture and facilities for work, in which they spend most of their working hours. In a narrow sense, this space could also be defined as a work desk. Thus, we assessed participants’ satisfaction with the room size (hereafter S_R_) and satisfaction with the desk size (hereafter S_D_) used for WFH during COVID-19 on a 5-point Likert scale ranging from 1 *=* extremely dissatisfied to 5 = extremely satisfied.

#### 2.2.2. Features of Home Workspace

Only physical and social environmental factors that could potentially impact telecommuters’ satisfaction with workspace size were selected as research variables. These variables can be divided into (1) factors directly associated with the objective size of the workspace and (2) factors indirectly affecting people’s perceptions of space size.

The former category includes variables determining the size of a home workspace or a house (i.e., home workspace size (m^2^), ceiling height (m), house size (m^2^), and the number of rooms). We also included variables related to spatial density, such as whether they shared the workspace or desk, the number of occupants, and the purpose of the home workspace. As for the purpose, we questioned whether the space was used only for work or for other purposes because we assumed that multi-function could affect occupants’ feelings of crowding.

The factors indirectly influencing satisfaction with space size were selected from the literature review. We constructed variables by establishing six categories: physical openness [21], visual openness [49], lighting [52,53], noise [50], visual distractions [55,56], and violation of privacy [57]. Specific environmental variables for each category (see Table 1) were determined in consideration of the context of the WFH environment while referring to previous studies. Table 1 lists the environmental variables for each category.

#### 2.2.3. Ratings for the Home Workspace

The design and use of the workspace impact not only workers’ perceptions of environments but also their job performance and work engagement [72,73]. Describing that “these are the cornerstones of the domain known as the environmental psychology of workspace,” Vischer developed a comprehensive model, including various dimensions for designing a functionally comfortable workspace and goals to achieve [73].

Among the suggested dimensions, we extracted aspects suitable for the WFH context (i.e., functional comfort, psychological comfort, physical comfort, physiological comfort, resilience, individuality) and translated the terms to help the survey respondents understand their meanings more easily (i.e., concentration, attachment to home, ergonomic comfort, physiological comfort, relieving stress, and privacy). Then, 5-point Likert scale questions were created to measure satisfaction with the home workspace in each dimension. Regarding concentration, for example, respondents were asked, “How satisfied are you with your home workspace in terms of work concentration?” We also included a question about general satisfaction with the WFH environment.

#### 2.2.4. Change in Health and Productivity

Vischer presented workers’ well-being, productivity, and health (physical, mental, and social) as goals of a functionally comfortable workspace [73], which became the criterion for establishing the main dependent variables of this study. We replaced well-being with work–life balance since well-being, defined as the state of feeling healthy and happy [74], can be confused with health. We also considered perceived differences in health and productivity before and after the pandemic to measure changes due to WFH. Thus, we established five dependent variables: perceived changes in physical health, mental health, work–life balance, social well-being, and productivity.

As for physical health, we asked participants about three items: overall physical health, drowsiness while working, and sleep quality during each period of working in the office “before” the pandemic and working from home “after” the pandemic. The change in each item was obtained by subtracting “before” from “after.” The change in physical health was obtained by calculating the average value of the three items, and mental health was measured by overall mental health, depression, and stress while working. Productivity included six items, which are overall productivity, job satisfaction, work engagement, work enjoyment, work energy, and work concentration [75]. Table 2 describes how the dependent variables were calculated from the data obtained.

#### 2.2.5. Control Variables

To control for respondents’ demographic characteristics, the survey included questions about age, sex, marital status, place of residence, education, employment status, housing type, and children at home. We also investigated the characteristics of their WFH experience, including the frequency of WFH per week, daily working hours, prior experience of WFH, and the compulsoriness of WFH. In multiple linear regression analyses, we incorporated these variables as predictors.

### 2.3. Analytic Approaches

To test our first hypothesis, Spearman’s rank correlation analysis and an independent samples T-test were performed. The second and third hypotheses were tested by conducting a series of multiple linear regression analyses using a stepwise method to find significant predictors for outcome variables, including ratings for home workspaces and changes in health and productivity. SPSS version 28 was used for these analyses.

## 3. Results and Discussion

### 3.1. Environmental Features and Feelings of Crowding (H1)

#### 3.1.1. Space Size and Density

Table 3 presents the results of the correlation analyses and independent T-tests of size-related environmental variables and S_R_/S_D_. Interestingly, S_R_ significantly correlated with the size of the entire house (*ρ* = 0.35, *p* = 0.000), not with the size or ceiling height of the home workspace. This result is consistent with past research that found a significant association between office size and crowding feelings [18,22], but it raises a question regarding telecommuters’ perceptions of occupied space in the WFH context.

Personal space in the office can be defined as the region that others cannot enter without causing discomfort [76]. Therefore, the amount of personal space depends on who these others are, how close they are to the subject, and whether they are always there. These social aspects may distinguish WFH environments from offices. A more extensive investigation is required to determine the perceived range of workspaces in a WFH setting, but this study suggests that respondents may have regarded the entire house as a space they occupied or used while WFH. Thus, telecommuters’ S_R_ may be more strongly correlated with the total area they can freely occupy than the space they actually use during work hours.

The number of rooms was also significant (workspace size: *ρ* = 0.26, *p* = 0.000; desk size: *ρ* = 0.19, *p* = 0.002). The number of rooms is likely to determine whether a telecommuter can have a dedicated or private room for work, and the chance of having a dedicated or private room increases S_R_ and S_D_. The results of the independent T-test in Table 2 reflect this. Sharing the home workspace did not cause a significant difference in satisfaction, but the space functions made a significant difference in S_D_ (*t* = −3.85, *p* = 0.005). Although we have not investigated what the other functions were, it can be assumed that multifunctional home workspaces would have prevented telecommuters from having fully dedicated and well-organized home office desks. This result supports recent studies—e.g., [77,78,79]—that stress the importance of a dedicated workspace for WFH.

Contrary to our expectations, people living with others rated higher S_R_ than those living alone (*t* = 2.52, *p* = 0.012). To take a closer look at this result, we compared workspace size, house size, and number of rooms between these two groups. Table 4 shows that although workspace size was not significantly different, the house size when living with others was twice that when living alone (*t* = 8.97, *p* = 0.000), and the number of rooms was almost double (*t* = 9.64, *p* = 0.000). These results, consistent with Table 3, confirm that house size and the number of rooms played a more decisive role in S_R_ than workspace size itself.

#### 3.1.2. Physical and Visual Openness

Table 5 presents the results for visual and physical openness. The variables indicating visual openness, including the number of windows and the presence of a front window in the home workspace, were not associated with S_R_ or S_D_. In contrast, the presence of an accessible balcony or domestic garden, which seemed to be related to the physical openness of a house, made people happier with the WFH room size (*t* = 2.01, *p* = 0.043). Numerous recent studies have revealed the health benefits of balconies or home gardens during the COVID-19 lockdown—e.g., [80,81]. Interestingly, however, our data showed no significant association between the frequency of using a balcony/garden while working and S_R_/S_D_. Furthermore, nearly 40% of respondents with a balcony/garden answered that they never used this space, even in summer, while working from home.

These findings suggest that, even if a particular space is actually rarely used, whether the space is given to users or not can affect their satisfaction. This inference is in line with the discussion mentioned above that the perception of workspace size tends to depend on the amount of space they can comfortably occupy (without the feeling of intruding on someone else’s space) rather than the space size they actually use. Furthermore, this may imply that satisfaction with space size is more related to psychological factors, such as individual control or autonomy over the use of space, than to the amount of space actually used. Related to this, Knight and Haslam studied the effects of allowing employees to decorate the office by themselves. Their work showed that this empowerment, what they termed the “empowered condition”, improved the well-being and productivity of workers [82]. Our results, along with their findings, suggest a potential research topic on the psychological aspects that affect telecommuters’ awareness and appraisal of the WFH environment, health, and productivity. It also supports the rationale for activity-based offices, which have recently been gaining popularity worldwide, where workers have the autonomy to choose a workspace among varied workspace types [83].

#### 3.1.3. Lighting, Noise, and Violation of Privacy

Table 6 shows that all lighting- and noise-related variables were significantly correlated with S_R_. In particular, the quality of light was strongly linked to S_R_ (*ρ* = 0.41, *p* = 0.000) and S_D_ (*ρ* = 0.14, *p* = 0.023), which is in line with previous studies [52,54]. Both the frequency of noise from outside (*ρ* = 0.25, *p* = 0.000) and inside (*ρ* = 0.14, *p* = 0.024) were associated with S_R_. This result is consistent with prior research showing that noise disturbance lowers office workers’ environmental satisfaction [84]. This result is particularly intriguing because the mean values of noise frequency both from outside (*M* = 4.04) and inside (*M* = 4.14) were low (a 5-point Likert scale ranging from 1 = always to 5 = never), suggesting that even infrequent noise can significantly affect telecommuters’ perceptions of the home workspace size.

Only acoustical privacy was linked with S_R_ (*ρ* = 0.21, *p* = 0.001) and S_D_ (*ρ* = 0.13, *p* = 0.044). Considering that privacy is one of the paramount issues in the work environment [85], the insignificant result of visual privacy is somewhat unexpected. This result can be explained by the social aspects of the WFH environment. In a typical work environment, where workers are usually surrounded by colleagues, they can be more sensitive to situations in which other people can see them unexpectedly or where they are forced to see things they do not want to see. In contrast, people may be less sensitive to or annoyed by these visual privacy invasions in their home workspaces because they usually share space with the family.

On the other hand, it seems that the reason why acoustic privacy violations have serious consequences is that, as already discussed, even infrequent noises have a significant impact on telecommuters’ perceptions of the WFH environment. In addition, the sound is usually harder to block or control than sight. Accordingly, regardless of the type of work environment, people may feel uncomfortable if they generate noise that can bother others (even family members) or if they are exposed to noise (even from family members).

#### 3.1.4. Visual Distractions

As shown in Table 7, the more positively they evaluated the aesthetic pleasure of the home workspace (*ρ* = 0.37, *p* = 0.000) and the scenery through the window (*ρ* = 0.32, *p* = 0.000), the more satisfied the respondents were with the WFH room size. In particular, when greenery was included in the scenery, the respondents were significantly more satisfied with workspace size (*t* = −3.33, *p* = 0.001). On the other hand, there was no significant result for the presence of plants, which was surprising considering the numerous studies demonstrating the health and emotional benefits of indoor plants in the workplace [86,87].

Thus, we further investigated whether the presence of plants affected WFH environment ratings and changes in health and productivity. The T-test results showed that the attachment to home was significantly greater with indoor plants (*M* = 4.06) than without (*M* = 3.78) in the home workspace (*t* = −2.57, *p* = 0.011). The presence of art objects showed exactly the same result in that it made no significant difference in S_R_ or S_D_, but in the attachment to home (*t* = −2.35, *p* = 0.02). Although it was not possible to determine whether plants and art objects enhanced occupants’ attachment to the home or whether strong attachments increased the desire to decorate their home, at least we could confirm that emotional intimacy with home was related to the presence of plants and artworks.

### 3.2. Ratings for the Home Workspace (H2)

**Figure 2:**
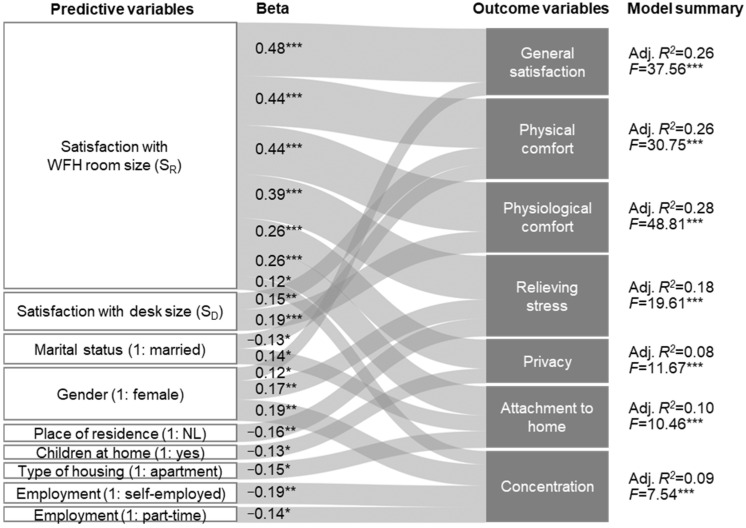
Regression models predicting ratings for home workspace (* *p* < 0.05, ** *p* < 0.01, *** *p* < 0.001).

We conducted multiple linear regression analysis using a stepwise method, with each home workspace rating as an outcome variable. Figure 2 shows the seven regression models schematized as the Sankey diagram. To create a Sankey plot showing a regression model, we referred to the study of Neff et al. [88]. Appendix A includes a table of regression models. In Figure 2, predictive variables are presented on the left, and the width of the ribbon coming from each predictor represents the size of the standardized coefficients (beta), regardless of their polarity. Seven outcome variables are shown on the right, with a summary of each model.

It was found that S_R_ significantly predicted all outcome variables. Additionally, S_D_ had a significant effect on physical and physiological comfort in the WFH environment. These results confirm our second hypothesis, suggesting that satisfaction with workspace size positively affects how occupants assess their residential work environments. In addition, this suggests that the significant effect of crowding feelings on workplace satisfaction in an office environment identified in the previous study [20] can also be applied in the context of WFH.

Women were more satisfied with the WFH environment than men in terms of general satisfaction, relieving stress, and concentration. This result is somewhat surprising, considering that women’s housework hours have increased, and employment instability has worsened during the lockdown in Korea [89]. However, it becomes quite understandable considering the early days of the pandemic in Korea when children were forced to take online classes at home, although WFH had not yet been activated. At that time, many parents (especially working moms) with young children had to struggle between parenting and working [90], which might have made them favor the WFH recommendations and positively affected their satisfaction with the WFH environment.

Having children at home had a negative effect on privacy satisfaction, which is consistent with Pluut and Wonders, who found that people with children experienced more deterioration in blurred work–life boundaries during the pandemic [4]. Self-employed and part-time workers reported lower satisfaction with concentration than full-time workers. This may reflect the fact that overall employment insecurity increased during the pandemic [89,91]. The anxiety induced by employment insecurity may have negatively affected the perceived level of concentration within the WFH environment.

### 3.3. Changes in Health and Productivity (H3 & H4)

**Figure 3:**
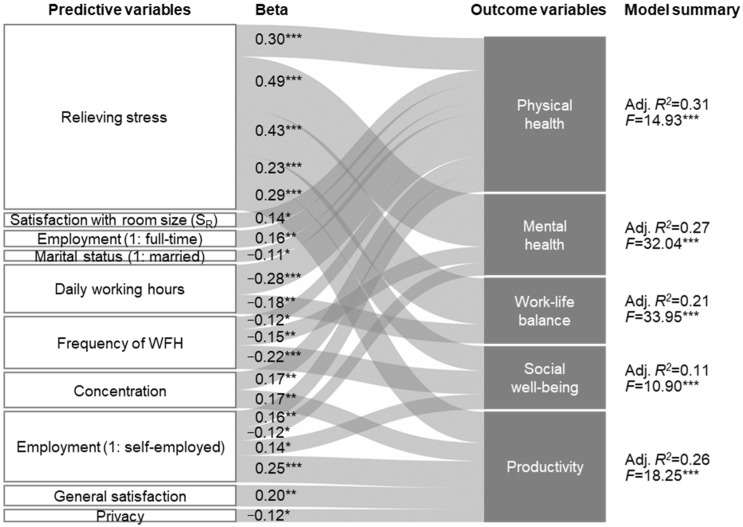
Regression models predicting changes in health and productivity (* *p* < 0.05, ** *p* < 0.01, *** *p* < 0.001).

Figure 3 presents five regression models (also shown as a table in Appendix A) predicting changes in health and productivity before and during COVID-19.

Relieving stress had a very strong positive effect on all outcome variables, suggesting that creating a WFH environment that reduces stress is paramount to maintaining telecommuters’ health and productivity. This result can be further supported by Seva et al.’s discussion that the WFH environment is more likely to be associated with unpleasant feelings and stress than the office environment [11]. Additionally, we found significant effects of concentration and general satisfaction on physical health and productivity, which supports our fourth hypothesis (H4a, b), arguing that home workspace ratings would have affected changes in health and productivity. In contrast, regarding our third hypotheses (H3a, b), S_R_ or S_D_ was not evident for the outcome variables except for physical health. However, since we have already demonstrated that S_R_ strongly affects how people evaluate their home workspace, it can be inferred that such satisfaction with the space size will indirectly impact the health and productivity of telecommuters.

Meanwhile, it was found that daily working hours negatively affected physical health and work–life balance. Similarly, WFH frequency had a negative impact on physical/mental health and social well-being. The finding that social well-being deteriorates as WFH occurs more frequently demonstrates the physical and psychological isolation that too frequent WFH can bring. This outcome highlights the need for a balance between office-based and home-based work, such as hybrid work. In addition, it requires us to think about new forms of office and residential space that can better cope with the changing work landscape in response.

### 3.4. Contributions and Practical Implications

Our work contributes to the literature on the interactions between work environments and worker responses. More specifically, this study improves our understanding of the WFH environment and telecommuters’ perceptions of home workspace size. In particular, this study presented an interesting discussion of how dissimilarities in terms of the physical and social contexts of the workplaces (i.e., office versus home) make differences in workers’ responses to environmental factors. For example, while the impacts of light and noise on worker satisfaction appeared to be consistent between the office and home, responses to privacy intrusion in the telecommuting environment were found to be different than in the office environment. Additionally, the results of this study implied a possible difference in the perceived spatial extent of workspace between telecommuters and office workers. This implies that differences in the psychological or emotional connection a person has to space can create inherent differences in the mechanisms by which environmental factors influence behaviors, which will provide an important clue for future studies comparing WFH and office work.

This study shares the same ultimate goal (i.e., providing a healthy and productive WFH environment) as recent studies examining the effects of WFH environments on telecommuters’ behavioral and emotional responses—e.g., [6,9,11]. However, to the best of our knowledge, no studies have investigated factors affecting telecommuters’ feelings of crowding and its effects on their health and productivity, which is what makes our findings novel and potentially important.

In this paper, we demonstrated how various environmental features of home workspaces (e.g., house size, purpose of workspace, accessible balcony, lighting, noise, privacy, aesthetic pleasure, and scenery through windows) significantly affected telecommuters’ S_R_ or S_D_. In addition to physical environmental factors, S_R_ was found to be associated with psychological aspects, such as individual control or autonomy over space use. We believe that these results are essential in developing strategies to create a healthy and effective WFH environment. This is because, as we found through testing the second and third hypotheses, feelings of crowding can directly and indirectly affect telecommuters’ satisfaction with their work environments, as well as their well-being and work performance.

This research also offers various potential ways to alleviate crowding issues in the WFH environment without increasing the workspace size given to telecommuters. First, it would be advantageous to have a separate workspace dedicated to working. Even if the workspace is shared with others, the crowding issue can be alleviated if the space is used only for work. This suggestion could also contribute to resolving the issue of blurred work–life boundaries, which many researchers have pointed to as one of the biggest challenges that telecommuters should overcome [2,3,4].

Second, light quality in the home workspace is one of the most important factors affecting telecommuters’ feelings of crowding. Satisfaction with the home workspace size can increase if sufficient natural light is introduced and the need for artificial lighting is low. In addition, it is well known that light directly affects an individual’s physical and mental health [92]. Therefore, it would be beneficial to place the workstation in a room with as much natural light as possible.

Third, our results have shown that even infrequent noise (especially from outside) can make telecommuters feel crowded. Numerous studies have shown the harmful consequences of continuous noise exposure on workers’ health—e.g., [84,93,94]. Although it cannot be said that residential buildings are generally more susceptible to noise than typical office buildings, noise problems between floors and walls in apartments are constantly being pointed out [95,96,97]. Additionally, the natural ventilation that most houses rely on may make it difficult to control outside noise. To create healthy and efficient home-based work environments, residential building designs should pay greater attention to noise issues.

Fourth, pleasant visual stimuli, such as the aesthetic pleasure of the home workspace and satisfaction with the scenery through windows, appear to effectively alleviate the feeling of crowding in the home workspace. We also found that the presence of plants or artwork is associated with telecommuters’ home attachments. As Knight and Haslam showed, the beneficial effects of decorations in an office space [82] and creating a visually satisfactory home workspace can contribute to improving the psychological comfort, satisfaction, and productivity of telecommuters. According to a recently released Korean news report [98], more than half of 683 adult respondents said that their interest in organizing and decorating personal space at home had increased since COVID-19 began. The biggest reasons for the increased interest were “to put my favorite items on the desk,” “to relieve stress and take psychological comfort,” and “because my time at home increased.”

Lastly, our results showed that the presence of balconies mitigated the feeling of crowding in the home workspace, although the actual frequency of using balconies was not very high. Additionally, not the home workspace size but the entire house size was found to be correlated with home workspace size satisfaction. These results suggest that the perceived size of personal space (even if it is rarely used) rather than the amount of space actually used plays a more decisive role in the ratings for the space size. To convert these results into practical design ideas, further research is required on how the spatial range of personal space perceived by telecommuters varies according to various physical and social living environment conditions.

We believe that these suggestions for alleviating feelings of crowding in the home workspace without increasing the amount of workspace are important because it is usually impossible or very difficult to provide additional space in an existing building. During the pandemic, many novice telecommuters had to struggle to fit their workspace into the existing physical space of their home, which was already crammed with other domestic functions. Therefore, we expect that providing them with several ideas that can be applied without changing the structure of the building will be useful for them to avoid feeling crowded, even in an insufficient amount of space. Furthermore, some of the ideas discussed above will provide insights into improving the design of residential spaces in the future to better accommodate a new type of work.

### 3.5. Limitations and Future Research

Several potential limitations need to be considered. First, although changes in health and productivity were considered dependent variables, we were not able to conduct a longitudinal study. Instead, we asked survey respondents to recollect the status of their health and productivity before the pandemic, which might lead to biased or inaccurate answers—a memory bias. Second, we recruited Korean participants living in Korea and the Netherlands to consider the various types and conditions of residential environments. Although Korea and the Netherlands are similar in that they both have advanced ICT, which is a prerequisite for WFH, the fact that only the Netherlands was included as a country other than Korea may hinder the generalization of the research findings. Third, since the number of independent variables was too large, we decided to use bivariate analysis to test the first hypothesis. This method reveals the correlation between variables but has a limitation in that it cannot confirm the causal relationship between variables. The results of this study can be supplemented by using a structural equation model that integrates all hypotheses with more simplified variables. Finally, participants for the online survey were recruited through social networking services, which means that the sample was composed of those who had internet access and had sufficient interest in this study. Additionally, the number of samples may not be sufficient. Therefore, the representativeness of the sample may be limited, and the generalization of the findings should be made carefully.

This research has raised many questions in need of further investigation. First, this study presented an interesting assumption about the spatial scope of personal space perceived by telecommuters. Further studies on how the perceived range of home workspaces differs depending on home space layouts or cohabiting members will contribute to a better understanding of telecommuters’ experiences and perceptions. Second, the effects of environmental factors on telecommuters’ satisfaction and changes in their health and productivity were excluded from the scope of the study. Given that recent studies have provided varying results for these relationships—e.g., [6,9,11]—additional research on this topic may help us learn more about the link between a WFH environment and telecommuters’ responses. Third, we suggest that the degree of control that residents have over the use of living spaces may influence their perception of the WFH environment. The impact of this control has already been demonstrated in the office environment [82,99,100,101,102]. Loss of control in a WFH environment, where workers generally might have higher expectations of control than in a typical office environment, might lead to greater negative consequences. We expect that future studies of these psychological factors in the context of WFH will provide interesting implications, not only in the field of architectural planning but also in the field of environmental psychology research.

## 4. Conclusions

Given that WFH is now the new normal for workers all over the world, it is vital to understand how WFH environments affect telecommuters’ well-being and productivity. The current study aims to understand how the characteristics of home workspaces affected telecommuters’ emotional and behavioral responses during the COVID-19 pandemic and to provide design ideas for WFH environments. Particularly, the ‘feeling of crowding’, which is well known as a significant negative factor for the productivity and well-being of workers, was examined in the context of WFH. By conducting an online survey to investigate telecommuters’ feelings of crowding and the features of their home workspaces during COVID-19, this study confirmed significant relationships between various variables: the home workspace, crowding feelings, satisfaction, well-being, and work performance. The findings of this study highlight that crowding issues should be significantly considered when creating a healthy and effective WFH environment. Finally, we have suggested a variety of practical strategies to alleviate feelings of crowding in the WFH context by translating our findings into design ideas.

## Figures and Tables

**Table 1:** Variables of features of home workspace.

Categories	Environmental Variables ^1^	Type of Data	Type of Response
Space size	Workspace size	Continuous	Open-ended
	Ceiling height of workspace		
	House size		
	No. of rooms	Discrete	
Density	Frequency of sharing of desk ^2^	Ordinal	Likert scale
	Sharing of workspace	Nominal	Multiple-choice
	Living alone		
	Purpose of workspace		
Physical openness	Accessible balcony/garden	Nominal	Multiple-choice
	Frequency of using balcony/garden	Ordinal	Likert scale
Visual openness	Presence of front window	Nominal	Multiple-choice
	No. of windows	Discrete	Open-ended
Lighting	Quality of light	Ordinal	Likert scale
	Sufficiency of natural light		
	Need for artificial light ^2^		
Noise	Frequency of noise inside ^2^	Ordinal	Likert scale
	Frequency of noise outside ^2^		
Violation of privacy	Violation of visual privacy ^2^	Ordinal	Likert scale
	Violation of acoustical privacy ^2^		
Visual distractions	Aesthetical pleasure	Ordinal	Likert scale
	Cleanliness		
	Rating for scenery		
	Type of scenery	Nominal	Multiple-choice
	Presence of plants		
	Presence of art objects		

^1^ All variables were set in favor of WFH as scores increased. Accordingly, the frequency of the sharing desk and noise inside/outside were measured on a 5-point Likert scale, with higher scores indicating less frequency (1 = always and 5 = never). The need for artificial light and the violation of visual/acoustical privacy were set to decrease as the score increased. ^2^ The scores of these variables mean the opposite of what the variables represent.

**Table 2:** The process of calculating changes in health and productivity.

Dependent Variables		Questions		Change ^1^ between before and after		Cronbach’s Alpha
		before	after			
Physical health	Overall	(1)	(2)	(1)–(2)	Average	0.62
	Drowsiness	(3)	(4)	(3)–(4)		
	Sleep quality	(5)	(6)	(5)–(6)		
Mental health	Overall	(7)	(8)	(7)–(8)	Average	0.77
	Depression	(9)	(10)	(9)–(10)		
	Stress	(11)	(12)	(11)–(12)		
Work–life balance		(13)	(14)	(13)–(14)		-
Social well-being		(15)	(16)	(15)–(16)		-
Productivity	Overall	(17)	(18)	(17)–(18)	Average	0.85
	Job satisfaction	(19)	(20)	(19)–(20)		
	Engagement	(21)	(22)	(21)–(22)		
	Enjoyment	(23)	(24)	(23)–(24)		
	Energy	(25)	(26)	(25)–(26)		
	Concentration	(27)	(28)	(27)–(28)		

^1^ Negative and positive numbers mean deterioration and improvement in health or productivity, respectively.

**Table 3:** Relationships between size-related environmental features and satisfaction with home workspace size.

Analysis	Environmental Features		Satisfaction			
			Room Size (S_R_)		Desk Size (S_D_)	
Spearman’s Correlation	Workspace size (m²)		*ρ* = 0.05		*ρ* = −0.03	
	Ceiling height of workspace (m)		*ρ* = −0.02		*ρ* = −0.07	
	House size (m²)		*ρ* = 0.35 ***		*ρ* = 0.14	
	No. of rooms (ea)		*ρ* = 0.26 ***		*ρ* = 0.19 **	
	Frequency of sharing of desk		*ρ* = 0.20 **		*ρ* = 0.01	
Independent *t*-test	Sharing of workspace	Using by myself	*M* = 3.48	*t* = 0.72 *d* = 0.09	*M* = 2.83	*t* = 1.47 *d* = 0.19
		Sharing with others	*M* = 3.38		*M* = 2.69	
	Purpose of workspace	Working only	*M* = 3.46	*t* = −0.16 *d* = 0.02	*M* = 2.99	*t* = −2.85 ** d = 0.40
		Other activities	*M* = 3.44		*M* = 2.71	
	Living alone	Alone	*M* = 3.12	*t* = 2.52 * d = 0.38	*M* = 2.71	*t* = 0.79 *d* = 0.13
		With others	*M* = 3.53		*M* = 2.80	

* p < 0.05, ** *p* < 0.01, *** *p* < 0.001. *ρ* = Spearman’s correlation coefficient; *M* = Mean; *d* = Cohen’s d.

**Table 4:** Comparisons of those living alone and those living with others.

	Workspace Size (m²)			House Size (m²)			No. of Rooms (EA)		
	*M*	*t*	*d*	*M*	*t*	*d*	*M*	*t*	*d*
Living alone	18.81	−0.61	0.11	47.51	8.97 ***	1.46	1.54	9.64 ***	1.54
Living with others	17.25			93.86			2.99		

*** *p* < 0.001. *M* = Mean; *d* = Cohen’s d.

**Table 5:** Relationships between openness-related environmental features and satisfaction with home workspace size.

Analysis	Environmental Features		Satisfaction			
			Room Size (S_R_)		Desk Size (S_D_)	
Spearman’s Correlation	Frequency of using balcony/garden		*ρ* = −0.08		*ρ* = 0.02	
	No. of windows		*ρ* = −0.03		*ρ* = −0.05	
Independent *t*−test	Accessible balcony/garden	Yes	*M* = 3.56	*t* = 2.01 * d = 0.26	*M* = 2.83	*t* = 1.28 *d* = 0.17
		No	*M* = 3.28		*M* = 2.71	
	Presence of front window	Yes	*M* = 3.40	*t* = 0.42 *d* = 0.06	*M* = 2.92	*t* = −1.73 *d* = 0.25
		No	*M* = 3.46		*M* = 2.74	

* *p* < 0.05. *ρ* = Spearman’s correlation coefficient; *M* = Mean; *d* = Cohen’s d.

**Table 6:** Relationships between lighting, noise, and privacy and satisfaction with home workspace size.

Analysis	Environmental Features	Satisfaction	
		Room Size (S_R_)	Desk Size (S_D_)
Spearman’s Correlation	Quality of light	*ρ* = 0.41 ***	*ρ* = 0.14 *
	Sufficiency of natural light	*ρ* = 0.28 ***	*ρ* = 0.08
	Need for artificial light	*ρ* = 0.17 **	*ρ* = −0.00
	Frequency of noise inside	*ρ* = 0.14 *	*ρ* = 0.13 *
	Frequency of noise outside	*ρ* = 0.25 ***	*ρ* = 0.21 **
	Violation of visual privacy	*ρ* = 0.10	*ρ* = 0.11
	Violation of acoustical privacy	*ρ* = 0.21 **	*ρ* = 0.13*

* *p* < 0.05, ** *p* < 0.01, *** *p* < 0.001. *ρ* = Spearman’s correlation coefficient.

**Table 7:** Relationships between visual distractions and satisfaction with home workspace size.

Analysis	Environmental Features		Satisfaction			
			Room Size (S_R_)		Desk Size (S_D_)	
Spearman’s Correlation	Aesthetical pleasure		*ρ* = 0.37 ***		*ρ* = 0.26 ***	
	Rating for scenery		*ρ* = 0.32 ***		*ρ* = 0.09	
	Cleanliness		*ρ* = −0.08		*ρ* = 0.01	
Independent T-test	Type of scenery	With greenery	*M* = 3.62	*t* = −3.33 ** d = 0.43	*M* = 2.84	*t* = −1.50 *d* = 0.19
		Without greenery	*M* = 3.17		*M* = 2.70	
	Presence of plants	Yes	*M* = 3.52	*t* = −0.78 *d* = 0.10	*M* = 2.85	*t* = −1.11 *d* = 0.15
		No	*M* = 3.41		*M* = 2.74	
	Presence of art objects	Yes	*M* = 3.44	*t* = 0.08 *d* = 0.01	*M* = 2.79	*t* = 0.74 *d* = 0.01
		No	*M* = 3.45		*M* = 2.78	

** *p* < 0.01, *** *p* < 0.001. *ρ* = Spearman’s correlation coefficient; *M* = Mean; *d* = Cohen’s d.

## Data Availability

Not applicable.

## Appendix A

**Table A1:** Regression models (Beta coefficients) predicting ratings for the home workspace.

Predictors	Outcome Variables						
	General Satisfaction	Concentration	Ergonomic Comfort	Physiological Comfort	Relieving Stress	Attachment to Home	Privacy
S_workspace size	0.48 ***	0.12 *	0.44 ***	0.44 ***	0.39 ***	0.26 ***	0.26 ***
S_desk size			0.15 **	0.19 ***			
Gender (1: female, 0: male)	0.12 *	0.19 **			0.17 **		
Marital status (1: married, 0: not married)			−0.13 *			0.14 *	
Employment (1: self−employed, 0: employed)		−0.19 **					
Employment (1: part−time, 0: full−time)		−0.14 *					
Place of residence (1: Netherlands, 0: South Korea)					−0.16 **		
Type of housing (1: apartment, 0: other)						−0.15 *	
Children at home (1: yes, 0: no)							−0.13 *
*F*	37.56 ***	7.54 ***	30.75 ***	48.81 ***	19.61 ***	10.46 ***	11.67 ***
Adj. *R²*	0.26	0.09	0.26	0.28	0.18	0.10	0.08

** p* < 0.05, ** *p* < 0.01, *** *p* < 0.001.

**Table A2:** Regression models (Beta coefficients) predicting changes in health and productivity.

Predictors	Outcome Variables				
	Physical Health	Mental Health	Social Well-Being	Work–Life Balance	Productivity
Relieving stress	0.30 ***	0.49 ***	0.23 ***	0.43 ***	0.29 ***
Concentration	0.17 **				0.17 **
General satisfaction					0.20 **
Privacy					−0.12 *
S_workspace size	0.14 *				
Frequency of WFH	−0.12 *	−0.15 **	−0.22 ***		
Daily working hours	−0.28 ***			−0.18 **	
Employment (1: self−employed, 0: employed)	0.16 **	−0.12 *	0.14 *		0.25 ***
Employment (1: full−time, 0: part−time)	0.16 **				
Marital status (1: married, 0: not married)	−0.11 *				
*F*	14.93 ***	32.04 ***	10.90 ***	33.95 ***	18.25 ***
Adj. *R²*	0.31	0.27	0.11	0.21	0.26

** p* < 0.05, ** *p* < 0.01, *** *p* < 0.001.

## References

[1] Ammons S.K., Markham W.T. (2004). Working at Home: Experiences of Skilled White Collar Workers. Sociol. Spectr..

[2] Bouziri H., Smith D.R.M., Descatha A., Dab W., Jean K. (2020). Working from Home in the Time of COVID-19: How to Best Preserve Occupational Health?. Occup. Environ. Med..

[3] Cho E. (2020). Examining Boundaries to Understand the Impact of COVID-19 on Vocational Behaviors. J. Vocat. Behav..

[4] Pluut H., Wonders J. (2020). Not Able to Lead a Healthy Life When You Need It the Most: Dual Role of Lifestyle Behaviors in the Association of Blurred Work-Life Boundaries With Well-Being. Front. Psychol..

[5] Chung H., Seo H., Forbes S., Birkett H. (2020). Working from Home during the COVID-19 Lockdown: Changing Preferences and the Future of Work.

[6] Pang Z., Becerik-Gerber B., Hoque S., O’Neill Z., Pedrielli G., Wen J., Wu T. (2021). How Work From Home Has Affected the Occupant’s Well-Being in the Residential Built Environment: An International Survey Amid the Covid-19 Pandemic. ASME J. Eng. Sustain. Build. Cities.

[7] Holliss F. (2021). Working from Home. Built. Environ..

[8] D’Alessandro D., Gola M., Appolloni L., Dettori M., Fara G.M., Rebecchi A., Settimo G., Capolongo S. (2020). COVID-19 and Living Space Challenge. Well-Being and Public Health Recommendations for a Healthy, Safe, and Sustainable Housing. Acta Bio. Med. Atenei Parm..

[9] Araya Leon M.J., Abella A., Guasch R., Estévez A.T., Peña J. (2020). HETEROTOPIA WORK. Correlation between the Domestic Built Environment and Home Offices during COVID-19 Confinement. SDRJ.

[10] Vyas L., Butakhieo N. (2021). The Impact of Working from Home during COVID-19 on Work and Life Domains: An Exploratory Study on Hong Kong. Policy Des. Pract..

[11] Seva R.R., Tejero L.M.S., Fadrilan-Camacho V.F.F. (2021). Barriers and Facilitators of Productivity While Working from Home during Pandemic. J. Occup. Health.

[12] Evans G.W., Johnson D. (2000). Stress and Open-Office Noise. J. Appl. Psychol..

[13] Abdou O.A. (1997). Effects of Luminous Environment on Worker Productivity in Building Spaces. J. Archit. Eng..

[14] Wyon D.P. (2004). The Effects of Indoor Air Quality on Performance and Productivity: The Effects of IAQ on Performance and Productivity. Indoor Air.

[15] Akimoto T., Tanabe S., Yanai T., Sasaki M. (2010). Thermal Comfort and Productivity—Evaluation of Workplace Environment in a Task Conditioned Office. Build. Environ..

[16] Larsen L., Adams J., Deal B., Kweon B.S., Tyler E. (1998). Plants in the Workplace: The Effects of Plant Density on Productivity, Attitudes, and Perceptions. Environ. Behav..

[17] Haynes B.P. (2008). The Impact of Office Layout on Productivity. J. Facil. Manag..

[18] Oldham G.R. (1988). Effects of Changes in Workspace Partitions and Spatial Density on Employee Reactions: A Quasi-Experiment. J. Appl. Psychol..

[19] Allen T.J., Gerstberger P.G. (1973). A Field Experiment to Improve Communications in a Product Engineering Department: The Nonterritorial Office. Hum. Factors.

[20] Sundstrom E., Burt R.E., Kamp D. (1980). Privacy at Work: Architectural Correlates of Job Satisfaction and Job Performance. Acad. Manag. J..

[21] Charles K.E., Veitch J.A. (2002). Environmental Satisfaction in Open-Plan Environment: 2. Effect of Workstation Size, Partition Height and Windows.

[22] Dean L.M., Pugh W.M., Gunderson E.K.E. (1975). Spatial and Perceptual Components of Crowding: Effects on Health and Satisfaction. Environ. Behav..

[23] Fried Y., Slowik L.H., Ben-David H.A., Tiegs R.B. (2001). Exploring the Relationship between Workspace Density and Employee Attitudinal Reactions: An Integrative Model. J. Occup. Organ. Psychol..

[24] Oldham G.R., Fried Y. (1987). Employee Reactions to Workspace Characteristics. J. Appl. Psychol..

[25] International Labour Office (2016). Challenges and Opportunities of Teleworking for Workers and Employers in the ICTS and Financial Services Sectors: Issues Paper for the Global Dialogue Forum on the Challenges and Opportunities of Teleworking for Workers and Employers in the ICTS and Financial Services Sectors.

[26] Milasi S., González-Vázquez I., Fernández-Macías E. (2020). Telework in the EU before and after the COVID-19: Where We Were, Where We Head To.

[27] ICT Development Index. https://en.wikipedia.org/w/index.php?title=ICT_Development_Index&oldid=1093236130.

[28] Kim S., Ju J. (2014). Defining, Measuring, and Forecasting Telecommuting. Informatiz. Policy.

[29] Chau C. Majority of South Korean Businesses Opt for WFH Even after Pandemic. https://hrmasia.com/majority-of-south-korean-businesses-opt-for-wfh-even-after-pandemic/.

[30] Lim Y., Woo S., Lee S. (2022). Employees resist return to office in South Korea after 2 years of remote working—Pulse by Maeil Business News Korea. Pulse by Maeil Business News Korea.

[31] Lee C. (2022). More South Korean Firms Embrace Remote Working Arrangement. HRM Asia.

[32] Sundstrom E., Baum A., Epstein Y.M. (1978). Crowding as a Sequential Process: Review of Research on the Effects of Density on Humans. Human Response to Crowding.

[33] Kraut R.E., Fussell S.R., Brennan S.E., Siegel J. (2002). Understanding Effects of Proximity on Collaboration: Implications for Technologies to Support Remote Collaborative Work. Distributed work.

[34] Allen T.J. (1977). Managing the Flow of Technology.

[35] Allen T.J. (2007). Architecture and Communication among Product Development Engineers. Calif. Manag. Rev..

[36] Allen T.J., Henn G. (2007). The Organization and Architecture of Innovation: Managing the Flow of Technology.

[37] Becker F., Sims W. (2001). Offices That Work. International Workplace Studies Program.

[38] Gullahorn J.T. (1952). Distance and Friendship as Factors in the Gross Interaction Matrix. Sociometry.

[39] Hinds P.J., Kiesler S. (2002). The (Currently) Unique Advantages of Collocated Work. Distributed Work.

[40] Desor J.A. (1972). Toward a Psychological Theory of Crowding. J. Personal. Soc. Psychol..

[41] Veitch J.A., Charles K.E., Newsham G.R., Marquardt C.J.G., Geerts J. (2003). Environmental Satisfaction in Open-Plan Environments: 5. Workstation and Physical Condition Effects.

[42] Stokols D. (1976). The Experience of Crowding in Primary and Secondary Environments. Environ. Behav..

[43] Sinha S.P., Sinha S.P. (1991). Personal Space and Density as Factors in Task Performance and Feeling of Crowding. J. Soc. Psychol..

[44] (2010). Research and Design Recommendations on Crowding in Open-Plan Offices.

[45] Walden T.A., Nelson P.A., Smith D.E. (1981). Crowding, Privacy, and Coping. Environ. Behav..

[46] Aiello J.R., DeRisi D.T., Epstein Y.M., Karlin R.A. (1977). Crowding and the Role of Interpersonal Distance Preference. Sociometry.

[47] Six B., Martin P., Pecher M. (1983). A Cultural Comparison of Perceived Crowding and Discomfort: The United States and West Germany. J. Psychol..

[48] Fleishman L., Feitelson E., Salomon I. (2004). The Role of Cultural and Demographic Diversity in Crowding Perception: Evidence from Nature Reserves in Israel. Tour. Anal..

[49] Savinar J. (1975). The Effect of Ceiling on Personal Space. Man-Environ. Syst..

[50] Keeling T., Clements-Croome D., Roesch E. (2015). The Effect of Agile Workspace and Remote Working on Experiences of Privacy, Crowding and Satisfaction. Buildings.

[51] Dabbs J.M., Fuller J.P., Carr T.S. Personal Space When “Cornered”: College Students and Prison Inmates. Proceedings of the Annual Convention of the American Psychological Association.

[52] Mandel D.R., Baron R.M., Fisher J.D. (1980). Room Utilization and Dimensions of Density: Effects of Height and View. Environ. Behav..

[53] Schiffenbauer A., Aiello J.R., Baum A. (1979). Designing for High-Density Living. Residential Crowding and Design.

[54] Schiffenbauer A.I., Brown J.E., Perry P.L., Shulack L.K., Zanzola A.M. (1977). The Relationship between Density and Crowding: Some Architectural Modifiers. Environ. Behav..

[55] Baum A., Davis G.E. (1976). Spatial and Social Aspects of Crowding Perception. Environ. Behav..

[56] Worchel S., Teddlie C. (1976). The Experience of Crowding: A Two-Factor Theory. J. Personal. Soc. Psychol..

[57] Altman I. (1975). The Environment and Social Behavior: Privacy, Personal Space, Territory, Crowding.

[58] Baron R.A. (1994). The Physical Environment of Work Settings: Effects on Task Performance, Interpersonal Relations, and Job Satisfaction. Res. Organ. Behav..

[59] Kim J., de Dear R. (2013). Workspace Satisfaction: The Privacy-Communication Trade-off in Open-Plan Offices. J. Environ. Psychol..

[60] Dunstan J. (1979). The Effect of Crowding on Behaviour: Empirical Measures for Testing Theoretical Models. Urban Stud..

[61] Zoghbi-Manrique-de-Lara P., Sharifiatashgah M. (2019). The Emergence of Deviant Behaviors in the Physical Work Environment: A Study of Workers in Open Offices. IJM.

[62] Milgram S. (1970). The Experience of Living in Cities: Adaptations to Urban Overload Create Characteristic Qualities of City Life That Can Be Measured. Science.

[63] Saegert S. (1973). Crowding: Cognitive Overload and Behavioral Constraint. Environ. Des. Res..

[64] Stokols D. (1972). A Social-Psychological Model of Human Crowding Phenomena. J. Am. Inst. Plan..

[65] Baum A., Valins S. (1977). Architecture and Social Behavior: Psychological Studies of Social Density.

[66] Calhoun J.B. (1970). Population Density and Social Pathology. Calif. Med..

[67] Barker R.G., Gump P.V. (1964). Big School, Small School: High School Size and Student Behavior.

[68] VandenBos G.R. (2015). APA Dictionary of Psychology.

[69] Lusa S., Käpykangas S.M., Ansio H., Houni P., Uitti J. (2019). Employee Satisfaction With Working Space and Its Association With Well-Being—A Cross-Sectional Study in a Multi-Space Office. Front. Public Health.

[70] Kojima T., Sakuma T., Nishihara N., Hayashi T., Munakata J. (2017). Causal Modeling Between Workplace Productivity and Workers′ Satisfaction with Various Spaces in Office Buildings. J. Asian Archit. Build. Eng..

[71] (2020). 2019 Population and Housing Census.

[72] Vischer J.C., Fischer G.-N. (2005). User evaluation of the work environment: A diagnostic approach. Le Trav. Hum..

[73] Vischer J.C. (2008). Towards an Environmental Psychology of Workspace: How People Are Affected by Environments for Work. Archit. Sci. Rev..

[74] Cambridge Dictionary Well-Being (2020). Cambridge Advanced Learner’s Dictionary & Thesaurus.

[75] Pitchforth J., Nelson-White E., van den Helder M., Oosting W. (2020). The Work Environment Pilot: An Experiment to Determine the Optimal Office Design for a Technology Company. PLoS ONE.

[76] Ashkanasy N.M., Ayoko O.B., Jehn K.A. (2014). Understanding the Physical Environment of Work and Employee Behavior: An Affective Events Perspective: The physical work environment. J. Organiz. Behav..

[77] Xiao Y., Becerik-Gerber B., Lucas G., Roll S.C. (2021). Impacts of Working From Home During COVID-19 Pandemic on Physical and Mental Well-Being of Office Workstation Users. J. Occup. Envrion. Med..

[78] Leroy S., Schmidt A.M., Madjar N. (2021). Working from Home during COVID-19: A Study of the Interruption Landscape. J. Appl. Psychol..

[79] Awada M., Lucas G., Becerik-Gerber B., Roll S. (2021). Working from Home during the COVID-19 Pandemic: Impact on Office Worker Productivity and Work Experience. Work.

[80] Corley J., Okely J.A., Taylor A.M., Page D., Welstead M., Skarabela B., Redmond P., Cox S.R., Russ T.C. (2021). Home Garden Use during COVID-19: Associations with Physical and Mental Wellbeing in Older Adults. J. Environ. Psychol..

[81] Pouso S., Borja Á., Fleming L.E., Gómez-Baggethun E., White M.P., Uyarra M.C. (2021). Contact with Blue-Green Spaces during the COVID-19 Pandemic Lockdown Beneficial for Mental Health. Sci. Total Environ..

[82] Knight C., Haslam S.A. (2010). The Relative Merits of Lean, Enriched, and Empowered Offices: An Experimental Examination of the Impact of Workspace Management Strategies on Well-Being and Productivity. J. Exp. Psychol. Appl..

[83] Hoendervanger J.G., Van Yperen N.W., Mobach M.P., Albers C.J. (2022). Perceived Fit and User Behavior in Activity-Based Work Environments. Environ. Behav..

[84] Sundstrom E., Town J.P., Rice R.W., Osborn D.P., Brill M. (1994). Office Noise, Satisfaction, and Performance. Environ. Behav..

[85] De Croon E., Sluiter J., Kuijer P.P., Frings-Dresen M. (2005). The Effect of Office Concepts on Worker Health and Performance: A Systematic Review of the Literature. Ergonomics.

[86] Bringslimark T., Hartig T., Patil G.G. (2007). Psychological Benefits of Indoor Plants in Workplaces: Putting Experimental Results into Context. Horts.

[87] Dravigne A., Waliczek T.M., Lineberger R.D., Zajicek J.M. (2008). The Effect of Live Plants and Window Views of Green Spaces on Employee Perceptions of Job Satisfaction. Horts.

[88] Neff P., Simões J., Psatha S., Nyamaa A., Boecking B., Rausch L., Dettling-Papargyris J., Funk C., Brueggemann P., Mazurek B. (2021). The Impact of Tinnitus Distress on Cognition. Sci. Rep..

[89] Jones R.S. (2021). The Impact of the Pandemic on Women in the Korean Labor Market.

[90] Kim M.S. (2021). 86% of working moms and dads said, “The burden of work and childcare increases due to school closure and extended remote classes”. The Outsourcing Times.

[91] Kim S. South Korea’s Jobless Rate Hits 21-Year High as COVID Cases Rise. https://www.aljazeera.com/economy/2021/2/10/bb-southkoreasjobless-rate-hits-21-year-high-as-covid-cases-rise.

[92] LeGates T.A., Fernandez D.C., Hattar S. (2014). Light as a Central Modulator of Circadian Rhythms, Sleep and Affect. Nat. Rev. Neurosci..

[93] Banbury S., Berry D. (2005). Office Noise and Employee Concentration: Identifying Causes of Disruption and Potential Improvements. Ergonomics.

[94] Jahncke H., Hygge S., Halin N., Green A.M., Dimberg K. (2011). Open-Plan Office Noise: Cognitive Performance and Restoration. J. Environ. Psychol..

[95] Lee P.J., Kim Y.H., Jeon J.Y., Song K.D. (2007). Effects of Apartment Building Façade and Balcony Design on the Reduction of Exterior Noise. Build. Environ..

[96] Jeon J.Y., Ryu J.K., Lee P.J. (2010). A Quantification Model of Overall Dissatisfaction with Indoor Noise Environment in Residential Buildings. Appl. Acoust..

[97] Park S.H., Lee P.J., Yang K.S. (2016). Perception and Reaction to Floor Impact Noise in Apartment Buildings: A Qualitative Approach. Acta Acust. United Acust..

[98] Yoon G.I. (2021). A Growing Interest in Personal Space Due to COVID-19… Furniture Sales Exceeded 10 Trillion KRW. JKN News.

[99] O’Neill M.J., Carayon P. (1993). The Relationship between Privacy, Control, and Stress Responses in Office Workers. Proc. Hum. Factors Ergon. Soc. Annu. Meet..

[100] Lee S.Y., Brand J.L. (2010). Can Personal Control over the Physical Environment Ease Distractions in Office Workplaces?. Ergonomics.

[101] Lee S.Y., Brand J.L. (2005). Effects of Control over Office Workspace on Perceptions of the Work Environment and Work Outcomes. J. Environ. Psychol..

[102] Kwon M., Remøy H., van den Dobbelsteen A., Knaack U. (2019). Personal Control and Environmental User Satisfaction in Office Buildings: Results of Case Studies in the Netherlands. Build. Environ..

